# The Manganese Peroxidase Gene Family of *Trametes trogii*: Gene Identification and Expression Patterns Using Various Metal Ions under Different Culture Conditions

**DOI:** 10.3390/microorganisms9122595

**Published:** 2021-12-16

**Authors:** Yu Zhang, Zhongqi Dong, Yuan Luo, En Yang, Huini Xu, Irbis Chagan, Jinping Yan

**Affiliations:** Laboratory of Bioconversion, Life Science and Technology College, Kunming University of Science and Technology, No. 272 Jingming Road, Kunming 650500, China; yuzhangqbl@163.com (Y.Z.); dong201911805157@163.com (Z.D.); ly167102@163.com (Y.L.); yangenen82@yahoo.com (E.Y.); hnxusun@126.com (H.X.); Irbis@gmail.com (I.C.)

**Keywords:** white rot fungi, *Trametes trogii*, manganese peroxidase gene family, Mn^2+^, static culture

## Abstract

Manganese peroxidases (MnPs), gene family members of white-rot fungi, are necessary extracellular enzymes that degrade lignocellulose and xenobiotic aromatic pollutants. However, very little is known about the diversity and expression patterns of the MnP gene family in white-rot fungi, especially in contrast to laccases. Here, the gene and protein sequences of eight unique MnP genes of *T. trogii* S0301 were characterized. Based on the characteristics of gene sequence, all TtMnPs here belong to short-type hybrid MnP (type I) with an average protein length of 363 amino acids, 5–6 introns, and the presence of conserved cysteine residues. Furthermore, analysis of MnP activity showed that metal ions (Mn^2+^ and Cu^2+^) and static liquid culture significantly influenced MnP activity. A maximum MnP activity (>14.0 U/mL) toward 2,6-DMP was observed in static liquid culture after the addition of Mn^2+^ (1 mM) or Cu^2+^ (0.2 or 2 mM). Moreover, qPCR analysis showed that Mn^2+^ obviously upregulated the Group I MnP subfamily (*T_trogii*_09901, 09904, 09903, and 09906), while Cu^2+^ and H_2_O_2_, along with changing temperatures, mainly induced the Group II MnP subfamily (*T_trogii*_11984, 11971, 11985, and 11983), suggesting diverse functions of fungal MnPs in growth and development, stress response, etc. Our studies here systematically analyzed the gene structure, expression, and regulation of the TtMnP gene family in *T. trogii*, one of the important lignocellulose-degrading fungi, and these results extended our understanding of the diversity of the MnP gene family and helped to improve MnP production and appilications of *Trametes* strains and other white-rot fungi.

## 1. Introduction

Manganese peroxidases (MnPs), laccases (Lacs), and lignin peroxidases (LiPs) of white rot fungal strains are the most common extracellular ligninolytic peroxidases involved in lignocellulosic degradation [1]. MnPs and other lignin peroxidases degrade a wide range of natural and xenobiotic aromatic compounds due to lignocellulose’s complexity and random phenylpropanoid polymeric structure [1]. Thus, they have been widely used in various fields, including lignocellulose depolymerization [2], polymer synthesis, bio-bleaching of paper pulp [3], decolorization of textile dyes [4], biosensing [5], green chemistry [6], biotransformation, and detoxification of environmentally persistent aromatics [7].

MnPs are glycosylated heme-containing peroxidases with molecular masses ranging from 38 to 62.5 kDa (usually 330–370 amino acids) and contain a leader peptide of 21–29 amino acids, Mn^2+^-binding catalytic site, and some conserved domains such as N-terminal MAF and AAP [8,9,10]. MnPs can oxidize Mn^2+^ to Mn^3+^ and then the generated Mn^3+^ converts lignin phenolic compounds to phenoxy-radicals in the presence of chelators such as oxalate and malonate [1,2]. The catalytic cycle of MnP cleaves one molecule of H_2_O_2_ with the subsequent oxidation of the heme group within the enzyme structure [11]. MnP activity has been widely detected in wood-decaying white-rot fungi (e.g., *Agaricales*, *Corticiales*, *Polyporales*, and *Hymenochaetales*) and soil-littering decomposing fungi [4]. Moreover, white-rot fungi usually possess multiple MnP encoding genes, composing a gene family [9,12]. For example, there are ten and five MnP isoenzymes in *T. pubescens* strain FBCC735 [4] and *P. ostreatus* PODs [13], respectively.

Previous studies have shown that the growth conditions and culture media composition influence the gene expression and total enzyme activity of fungal MnPs [9,11,12,14]. For example, glycerol and walnut pericarp provided the highest MnP activity than other carbon sources (such as glucose, xylose, and sucrose) in *Cerrena unicolor*, one of the dominant enzymes producing strain, with the activity of 2.0 U/mL and 7.4 U/mL, respectively [15]. Moreover, the production of MnP was evidently stimulated by the addition of Mn^2+^ and aromatic compounds to the culture media [16], nitrogen limitation, growth on solid media instead of liquid cultures [15], white light [17], etc. Moreover, MnP enzyme yield and biochemical properties show a species-dependent and strain-dependent manner [14]. Those studies deepen our understanding of the factors affecting fungal MnP enzyme yield and the diversity of MnPs producing strains and gene family members, which provides opportunities for utilizing MnPs resources [14]. However, the widespread industrial use of fungal MnPs is limited partly because of the limited production of native MnP by white-rot fungi, low stability, and the lack of efficient heterologous expression systems, especially in contrast to fungal laccases [11,18].

Among white-rot fungi, strains belonging to *Trametes*, such as *T. pubescens* i8 [4] and *T. versicolor* BCC 775 [19], are outstanding producers of MnP, LiP, VP, and Lac [9,20,21]. Several purified MnPs, such as MnP TP55 from *T. pubescens* strain i8 and two MnPs (MnP1 and MnP2) from *T. polyzona* KU-RNW027, showed higher catalytic efficiency, organic solvent-tolerance or-activation, dye-decolorization ability, and detergent-compatibility than that of *Bjerkandera adusta* strain CX-9 (MnP BA30) and *Phanerochaete chrysosporium* [4,20]. Additionally, the available data from the whole genome sequence of *T. pubescens* strain FBCC735 [4], *T. trogii* S0301 [22], and *T. villosa* [23] demonstrated the diversity of the MnP gene family with ten, eight, and three isoenzymes, respectively. However, previous studies about *Trametes* strains mainly focused on laccase production ability with higher laccase productivity and their application [24]. In this study, we report the potential of *T. trogii* S0301, a thermotolerant strain, for MnP production. In order provide a comprehensive insight into the expression, regulation, and possible biological functions of the TtMnP gene family, the aims of this study were to (i) analyze *T. trogii* S0301 MnP gene family; (ii) unravel the expression patterns of different MnP isoenzymes and the dynamical changes of MnP activity; and (iii) explore the approaches to improve MnP production of *T. trogii* S0301.

## 2. Materials and Methods

### 2.1. Fungal Strains and Culture Conditions

The *T. trogii* S0301 (NCBI BioProject: PRJNA480364) strain was maintained on GYP medium (2% glucose, 0.5% yeast extract, 0.5% tryptone, and 0.1% MgSO_4_·7H_2_O) at 4 °C. All strains were stored at the Life Science Research Center of Biotechnology Research Center of Life Science and Technology College. Homogenized inocula of *T. trogii* S0301 were prepared according to the methods described in our previous studies [24].

For enzyme production and RNA preparation, the final concentrations of CuSO_4_ (0.2 and 2 mM), MnSO_4_ (0.5 and 1 mM), and H_2_O_2_ (0.1 and 5 mM) were added to the culture medium. The fungal hyphae of *T. trogii* S0301 were placed at 28 °C or 37 °C under static or shake at a rotary shaker with 160 rpm. The liquid cultures were sampled every 3 days, and the supernatants or hyphae were obtained by centrifugation at 8590× *g* for 5 min at 4 °C for further studies [25].

### 2.2. DNA and RNA Isolation and Cloning of Manganese Peroxidase Genes

According to the previous method [25], the total genomic DNA of the homokaryotic of *T. trogii* S0301 was extracted from 6-day cultured fresh fungal hyphae using the cetyltrimethylammonium bromide method. Extraction of total RNA and first-strand cDNA synthesis were performed using TRIzol reagents (Promega, Madison, WI, USA) and HiScript II Q Select RT Super Mix for qPCR (Vazyme Biotech, Nanjing, China). PCR analysis was performed using gene-specific primers (Table S1) to clone gene coding or promoter regions, and the resulting products were separated by agarose gel electrophoresis (0.8%) and visualized by ethidium bromide staining.

### 2.3. Manganese Peroxidase Activity Assay

MnP activity was measured according to the method of Angel et al. (2020) [4,26]. The final 1 mL volume of the reaction mixture contains 200 μL of the following components: 50 mM MES Buffer (pH 5), 1 mM MnSO_4_, 20 mM 2,6-DMP, 25 mM 4-aminoantipyrine, and an appropriate dilution of the enzyme solution. The reaction is initiated when 0.4 mM H_2_O_2_ was added, and the increase in absorbance at 510 nm was monitored at 40 °C every 30 s for three minutes. One unit of the enzyme activity was defined as the amount of enzyme that oxidized 1 μmol of the 2-6-DMP per min.

### 2.4. Real-Time PCR

According to genomic and transcriptome sequences [22], eight primers of manganese peroxidase genes for quantitative RT-PCR were designed (Table S2). The expression of manganese peroxidase gene was detected by an ABI Prism 7500 Fast real-time PCR system (Applied Biosystems, Foster City, CA, USA) [25]. Real-time PCR was performed using AceQ qPCR SYBR Green Master Mix (Vazyme Biotech, Nanjing, China). The amplification conditions were as follows: 94 °C (5 s) and 60 °C (30 s) for 40 cycles. The glyceraldehyde-3-phosphate dehydrogenase (*TtGpd*) gene of *T. trogii* S0301 was used as a constitutively expressed endogenous control, and the ΔΔ*C_T_* method was used to calculate the relative transcription level. All primers sets are shown in Table S2.

### 2.5. Sequence and Phylogenetic Analysis

The DNA sequences of MnPs were extracted from *T. trogii* S0301 genomic data and analyzed [22]. The ORFs of MnP genes were analyzed using the ORF Finder of NCBI (https://www.ncbi.nlm.nih.gov/ accessed on 14 October 2021). The gene structure of MnP was analyzed by online software Gene Structure Display Server (http://gsds.cbi.pku.edu.cn/ accessed on 14 October 2021). MEME (Multiple Expectation Maximization for Motif Elicitation, http://meme-suite.org/ accessed on 14 October 2021) and SMART (Simple Modular Architecture Research Tool, http://smart.embl.de accessed on 14 October 2021) were used to identify and annotate MnP domains [27]. Bioedit software was used for sequence alignment, and the software package MEGA 6 (http://www.megasoftware.net/ accessed on 14 October 2021) was used for phylogenetic analysis.

### 2.6. Data Analysis

The results of all experiments were based on the average of three independent experiments ± standard error representation. Statistical significance was determined by unpaired t-test and one-way Anova, and the significance level was set at 0.05 (* *p* < 0.05; ** *p* < 0.01; *** *p* < 0.001). A difference in *p*-value less than 0.05 was considered significant.

## 3. Results

### 3.1. Identification of the MnP Gene Family of T. trogii S0301

Eight members of the MnP multigene family (TtMnPs) were identified in the whole genome of the *T. trogii* S0301 strain based on the domains of heme binding and peroxidase activity (Figure S1). The deduced MnP proteins comprised 358–367 amino acids with a molecular weight of about 38.5 kDa. Theoretical isoelectric points (pI) of the eight MnP proteins ranged from 4.32 to 4.94. SignalP analysis shows that all MnPs have the signal peptide (Table S3), which means eight MnPs belong to secretory proteins.

### 3.2. Phylogenetic Analyses of the MnP Gene Family of T. trogii S0301

**Figure 1:**
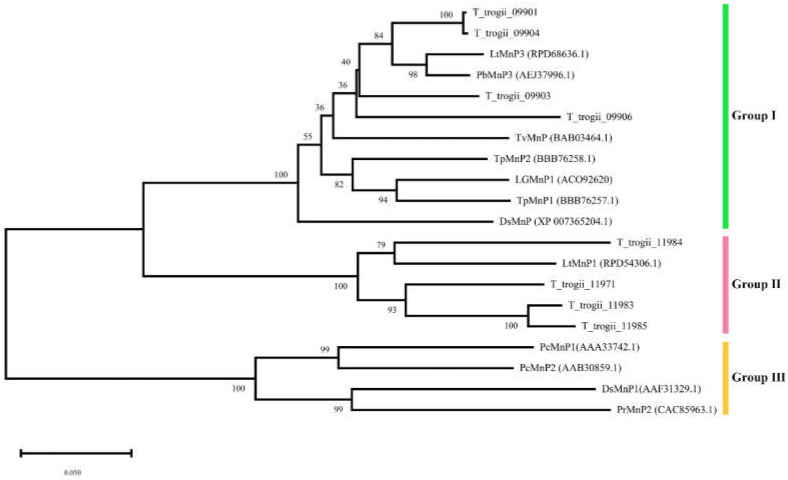
Maximum likelihood phylogenetic tree from TtMnPs and many related MnPs.

In order to further investigate phylogenetic relationships of TtMnPs, we constructed a neighbor-joining phylogenetic tree based on multiple sequence alignments of eight putative TtMnPs and other MnPs peptide sequences fungal strains, including *P. ostreatus*, *T. polyzona*, *P. chrysosporium*, and *Lentinula edodes* (Figure 1). According to the location on the phylogenetic tree, fungal MnPs were divided into three groups, and eight putative TtMnP clustered in the group I and II. *T_trogii_*09901, 09904, 09903, and 09906 were clustered in group I, and two MnPs of *T. polyzona* KU-RNW027 also belonged to this group, which were identified as short-type hybrid MnPs previously (Figure 1). The other four TtMnP (*T_trogii_*11984, 11971, 11985, and 11983) and LtMnP1 of *Lentinus tigrinus* were clustered within group II. However, no TtMnPs belonged to group III.

The analysis involved 20 MnPs from nine species containing *T. trogii* S0301, *L. tigrinus*, *Polyporus brumalis*, *T. versicolor*, *T. polyzona*, *T. gibbose*, *Dichomitus squalens* LYAD-421 SS1, *Phanerodontia chrysosporium*, and *Phlebia radiata*. Evolutionary analyses were conducted using MEGA6, and the bootstrap value was set as 1000 replicates, and the length of each branch is shown next to the branches. The numbers in the parentheses represent the sequence accession numbers in GenBank.

### 3.3. Gene Structure and Conserved Motifs of TtMnP Gene Family Members

**Figure 2:**
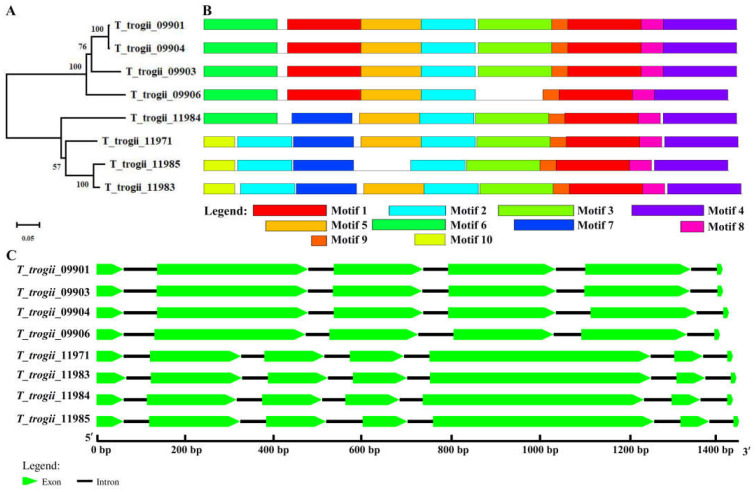
Phylogenetic analysis (**A**), conserved motifs (**B**), and exon–intron structure (**C**) of TtMnP gene family members.

**Figure 3:**
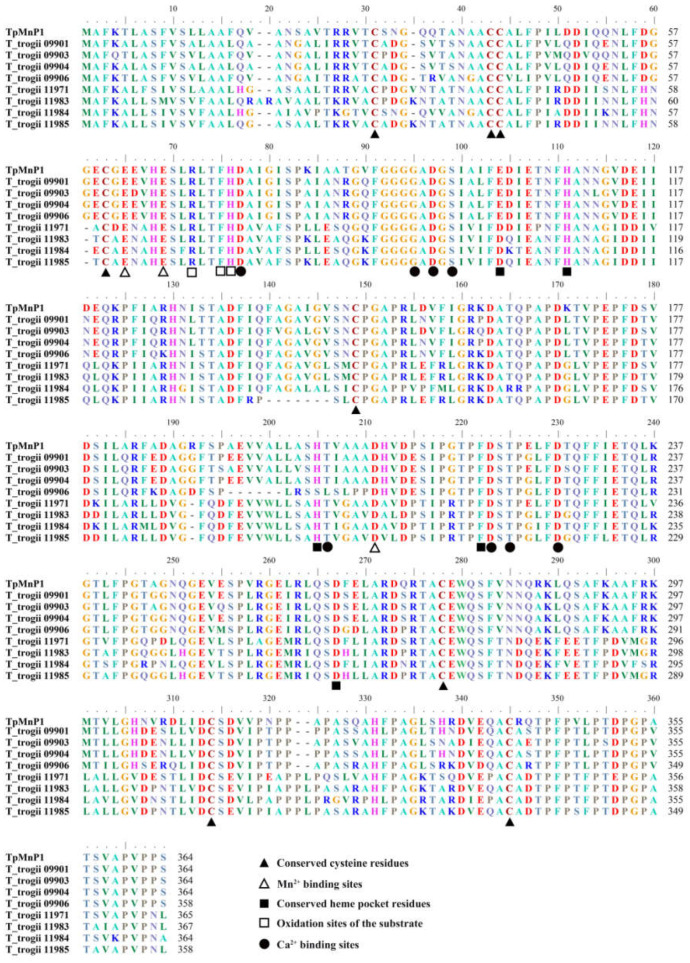
Multiple alignment of amino acid sequences of TtMnP gene family members and the distribution of catalytic and conserved amino acid residues of fungal MnPs.

In order to reveal the structural diversity of TtMnP gene family members, we constructed the exon/intron organizations and searched for conservative motifs. TtMnPs can be divided into two subfamilies (I and II) (Figure 2A). Group I contained *T_trogii_*09901, 09904, 09903, and 09906, and Group II contained *T_trogii_*11984, 11971, 11985, and 11983. The structural diversity of TtMnPs was investigated by identifying conserved motifs. The results showed that ten conserved motifs could be identified in TtMnPs, and the composition and location of those conserved motifs are some similarities, especially in the C-terminal domain (Figure 2B). For example, motifs 1, 2, 4, 7, 8, and 9 were found in all TtMnPs, which indicated that these conserved motifs were significant for the properties of manganese peroxidase. The deletion and duplication of some motifs were also detected. For example, *T_trogii_*11985 and 09906 do not contain motif 5 and motif 3, respectively. Moreover, motif 10 was mainly present in TtMnP belonging to Group II except for *T_trogii_*11984 (Figure 2B), suggesting functional divergence among some TtMnP. In addition, analyses of gene structures showed that TtMnPs commonly contained six to seven exons, and members of the same group had similar exon-intron structures (Figure 2C). Furthermore, all TtMnPs contained eight conserved cysteine residues, Mn^2+^ binding sites, conserved heme pocket residues, Ca^2+^ binding sites, and oxidation sites of the substrate, which are important catalytic and conserved amino acid residues of fungal MnPs (Figure 3).

### 3.4. Promoter of TtMnPs

**Figure 4:**
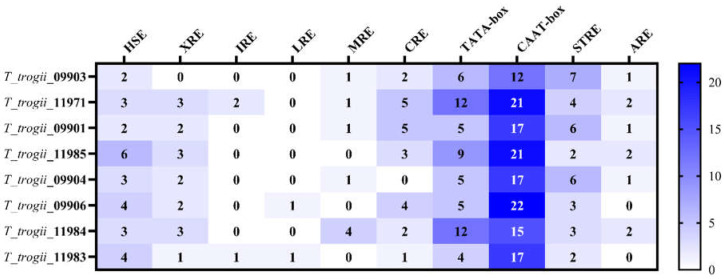
The number and the kind of the putitive responsive elements in the promoter regions of TtMnPs. Typical nucleotide sequences for each putative responsive element are as follows: HSE: GAANNTTC; XRE2:CACGCT XRE2:CACGCA; MRE2:TGCACCC MRE1:TGCGCGC MRE2:TGCGCCC; CreA2:CTGGGG CreA1:GCGGGG CreA3:CCGGGG CreA2:CCGGAG CreA1:GTGGAG; LRE1:CCGCCC; IRE1:CAGTGC; inverted CAAT BOX:ATTGG TATA BOX:TATAAA CAAT BOX:CAAT.

The distribution of the putative responsive elements in the 5′-flanking region of each TtMnP was analyzed. The promoter regions included various responsive element sequences [9], such as TATA and CAAT-box, heat shock element (HSE), xenobiotic response element (XRE), light-responsive element (LRE), metal responsive element (MRE), CreA-binding site (CRE), antioxidant responsive element (ARE), and a stress-responsive element (Figure 4). Moreover, the regulatory TATA and CAAT-box sequences, HSE and STRE are widely distributed in the promoter of all TtMnPs, with a total of 6–9 elements in each TtMnP promoter region (Figure 4). For example, there were four MREs in the promoter of *T_trogii_*11984, and no putative metal response elements were detected in *T_trogii_*11985, *T_trogii_*09906, and *T_trogii_*11983, while in the upstream region of other TtMnPs, one MRE motif was observed (Figure 4). In addition, we also observed the presence of XRE, CRE, and ARE.

### 3.5. Factors Affecting MnP Activity of T. trogii S0301

**Figure 5:**
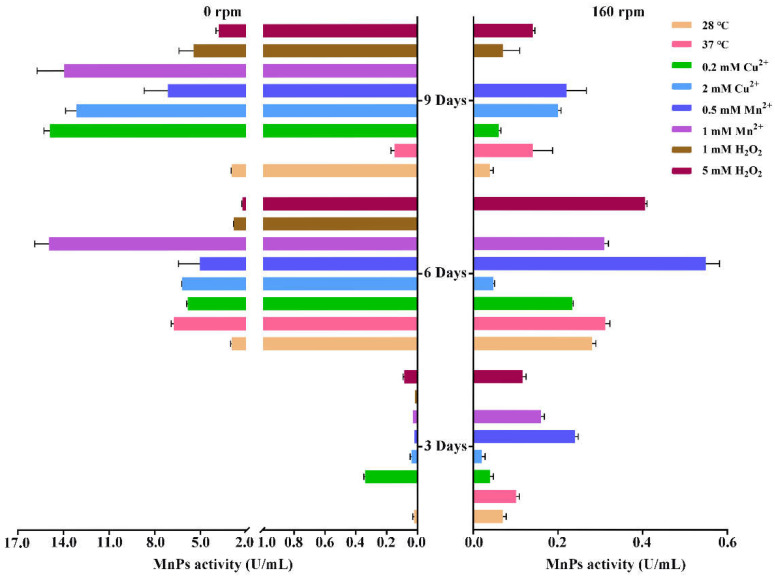
MnP production by *T. trogii* S0301 treated with Cu^2+^, Mn^2+^, and H_2_O_2_ and temperatures under static (**Left**) and shake (**Right**) liquid culture.

The influence of metal ions (Cu^2+^ and Mn^2+^) and H_2_O_2_ at different concentrations and temperatures (28 and 37 °C) on MnP production by *T. trogii* S0301 under the liquid culture was studied (Figure 5). Under static liquid culture, MnP activity appeared on day three. However, it showed maximum activity at day six with a maximum activity of about 16.5 U/mL when Mn^2+^ was added at the concentration of 1 mM. At day nine, the higher MnP activity (>5.0 U/mL) was detected under all conditions except at 37 °C (about 0.2 U/mL), and the maximum MnP activity (>14.0 U/mL) was obtained when Mn^2+^ (1 mM) and Cu^2+^ (0.2 and 2 mM) were added. In addition to Cu^2+^ and Mn^2+^, H_2_O_2_ at the concentration of 1 and 5 mM also stimulated the production of MnP with activities of 3.8 and 5.4 U/mL, respectively, compared to 2.9 U/mL for the control (28 °C). On the contrary, only slight MnP activity was detected under all conditions with shake culturing (160 rpm) (Figure 5), which is consistent with the results of our previous study [24]. In addition, only slight laccase activity (<0.3 U/mL) was detected under both static and shake liquid culture when Cu^2+^ was absent.

### 3.6. Expression Patterns of TtMnPs

**Figure 6:**
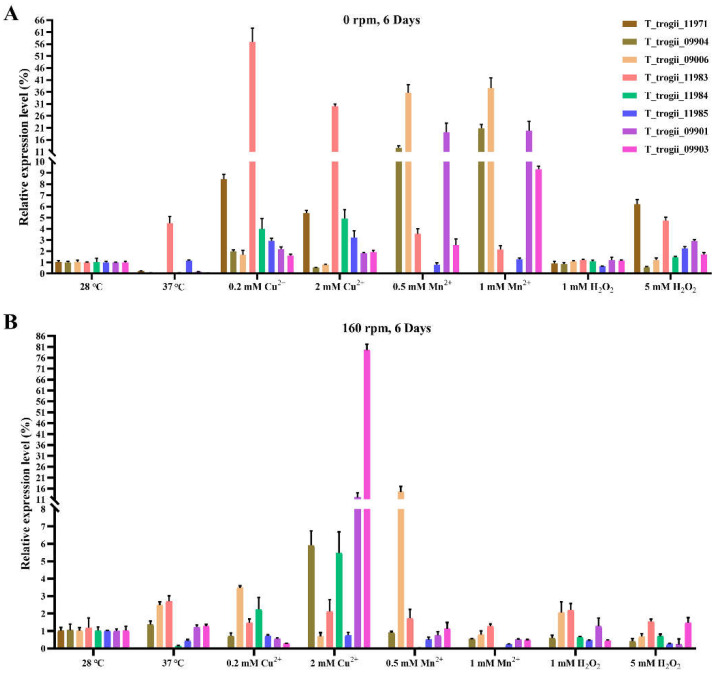
Gene expression patterns of MnPs in *T. trogii* S0301 treated with Cu^2+^, Mn^2+^, and H_2_O_2_ and temperatures under static (**A**) and shake (**B**) liquid culture by q-PCR.

In order to further explore the expression patterns of TtMnP under different treatments, the levels of TtMnPs transcripts were determined by q-PCR. The higher levels of TtMnPs transcripts were observed by Cu^2+^, Mn^2+^, H_2_O_2,_ and temperatures under static liquid culture than that of the shake culturing (Figure 6), which was in line with the change of MnP activity. Under static liquid culture, Mn^2+^ obviously upregulated the Group I MnP subfamily (*T_trogii_*09901, 09904, 09903, and 09906), while Cu^2+^, H_2_O_2_, and temperatures mainly induced the transcripts of the Group II MnP subfamily (*T_trogii_*11984, 11971, 11985, and 11983) (Figure 6A). The main resources of MnP activity under the static liquid culture at the presence of Cu^2+^ and Mn^2+^ were *T_trogii_*11983 and *T_trogii_*09906, respectively, with 51-fold and 31-fold induction compared to the control (28 °C). Under the shake liquid culture, gene transcription of the same MnP subfamily did not show a consistent pattern, and Cu^2+^ at the concentration of 2 mM showed the highest levels of MnP transcripts than compared to other treatments (Figure 6B). Cu^2+^ obviously upregulated three Group I MnPs (*T_trogii_*09901, 09904 and 09903) and one Group II MnP (*T_trogii_*11984) (Figure 6B).

## 4. Discussion

Gene family members of white-rot fungi possessing MnPs and laccases depicted different kinetic and physicochemical features, enhancing the possibility of new isoenzymes to lignocellulose and pollutants degradation [9]. However, the literature on the diversity and expression pattern of the MnP gene family in white-rot fungal strains is still limited, especially in contrast to laccases. Previous studies about MnP mainly focused on *P. chrysosporium* strains, which is thought to be the model MnP producing white-rot fungus [8]. However, other dominant wood rot fungi such as *Trametes* strains always are considered as laccase-producing strains. In the present study, the characteristics of the MnP gene family of *T. trogii* S0301 and the expression pattern of individual members were analyzed, which extends our understanding of the diversity of the MnP gene family and helps to improve MnP production in white-rot fungi.

White rot fungi are the primary source of lignin modifying enzymes, including laccases and MnPs. However, low production and low stability of fungal MnP are two major limiting factors that hampered their use on a large scale [11]. In recent years, multiple MnP encoding genes have been identified in non-model organisms due to genome projects and continued interesting research [22]. For example, there are ten and five MnP isoenzymes in *T. pubescens* strain FBCC735 [4] and *P. ostreatus* PODs [13], respectively. Here, we identified eight putative MnP genes from the genome of *T. trogii* S0301 strain (Figure 1). Based on the characteristics of gene sequence, MnPs can be divided into two types. Different MnP types are characterized by protein length, exon–intron pattern, and the number of disulfide bonds (four cystines) [28]. All TtMnPs of *T. trogii* S0301 here belong to type I with the average protein length of 363 amino acids, 5–6 introns, and the presence of conserved cysteine residues (Figure 2 and Figure 3; Table S3). However, whether *T. trogii* S0301 strain contains type II, MnPs remain uncertain.

Identifying the MnP gene family in several fungal strains is an opportunity for the development of MnP resources. However, fungal MnPs for industrial applications have still been limited due to the lack of an efficient genetic transformation system, allowing the manipulation of multiple genes in a suitable host [8]. In previous studies, many approaches such as heterologous expression of different MnP isozymes in plants [29], *Escherichia coli* [11], *Pichia pastoris*, and *Aspergillus* have been explored to increase MnP production. Nowadays, the most efficient method is improving the production of native MnP in white-rot fungi by optimizing enzymatic production conditions [29], including media composition, the addition of metal ions, incubation at different ranges of temperature or pH, the addition of Tween 80 or cofactors to the culture media, nitrogen limitation, and growth on solid media instead of liquid cultures [11]. The main factors altering MnP transcript and activity of white-rot fungi are summarized in Table 1.

Among those factors, Mn^2+^ is the most effective inducer of the gene expression and activity of MnP in *T. trogii* S0301 (Figure 5 and Figure 6), which is also detected in other *Trametes* strains (about 13-fold) when supplemented with Mn^2+^ at the concentration of 0.5 mM [21] and also in *T. pubescens* [4], *Pleurotus ostreatus* [10], *Ceriporiopsis subvermispora* [32], and so on (Table 1). Interestingly, Mn^2+^ at the concentration of 0.5 and 1 mM can selectively induce four TtMnPs belonging to group I, while showing a slight inhibition on group II TtMnP (Figure 5 and Figure 6) means that Mn^2+^ is a dual-directional regulation on MnP gene expression. In addition to Mn^2+^, Cu^2+^ [32], Fe^2+^ [21], and Se^2+^ [33] also affect gene expression and activity of MnP in fungal strains (Table 1). In this study, Cu^2+^ induced the transcripts of the Group II MnP subfamily (*T_trogii_*11984, 11971, 11985, and 11983), and the expression pattern is similar to that of H_2_O_2_ and temperatures (Figure 6A), which may suggest that the Group II MnP subfamily of *T. trogii* S0301 relates to fungi stress response. In addition, some studies suggested that laccase/MnP activity ratio can be regulated by adding both Cu^2+^ and Mn^2+^ at different concentrations [21]. Thus, the effects of both Cu^2+^ and Mn^2+^ on the gene expression and activity of MnP need to be explored further.

In addition to metal ions, we found that the static liquid culture also stimulated TtMnP gene expression. It resulted in higher enzyme activity (15 U/mL toward 2,6-DMP), but shake liquid culture is beneficial to the induction of hypha growth (Figure 5 and Figure 6), confirmed by MnP production in *Ganoderma boninense* with the highest MnP activity of 18–110 U/mL toward Mn^2+^ [30]. In our previous study with *T. trogii* S0301 strain, no MnP activity was detected in shake liquid cultures [24]. In addition, the MnP activity of many fungal strains is commonly lower when shake liquid cultures were employed (Table 1). Solid media favor MnP production than that of the liquid cultures [11]. Those results may suggest that the static culture is another crucial factor that affects gene expression and activity of MnP in white-rot fungi. The exciting thing is that a high laccase yield usually depends on the shake culture, while a high yield of MnP depends on the solid media or static culture. It remains uncertain whether the oxygen supply is the critical factor that maintains the balance between laccase and MnP during the static or shake culture conditions. Moreover, the biological function of MnP and laccase needs to be explored further.

In this study, we also found the distribution of putative responsive elements in the 5′-flanking regions of each TtMnP, such as ARE, XRE, HSE, and MRE, which means the complicated regulatory pattern of the MnP gene family responsive to biological and abiotic factors (Figure 4). Moreover, many other factors, such as heat shock, chemical stress, the type of lignocellulosic substrates, and nitrogen sources, appear to determine the types and amounts of ligninolytic enzymes produced by other white-rot fungi in a species-dependent or strain-dependent manner [14,35]. Therefore, further study is still needed to improve the production of MnP in white-rot fungi.

## 5. Conclusions

This study identified eight putative MnP genes from the genome of strain *T. trogii* S0301, all belonging to the type I MnPs. The expression patterns of the TtMnP gene family were analyzed using qPCR. The results showed that different expression patterns under the culture condition (static or shake) and under various stresses (Mn^2+^, Cu^2+^, H_2_O_2_, and temperature) combined with the presence of many putative responsive elements in the 5′-flanking regions of each TtMnPs, which indicatees diverse functions in growth, development, and stress response of fungal MnPs. Collectively, our study provides a comprehensive analysis and novel insights into the expression, regulation, and evolution of the TtMnP gene family in *T. trogii*, one of the important lignocellulose-degrading fungi; extends our understanding of the MnP gene family’s diversity; and helps improve MnP production and applications of *Trametes* strains and other white-rot fungi.

## Figures and Tables

**Table 1:** The factors altering MnP transcript and activity in fungi.

Strain	MnP Isoenzyme	Factor Up (↑) or Down (↓) Regulated	Culturecondition	MnP Activity (Substrate)	References
*G. boninense*	Gb_U6011	Nitrogen source ↑ H_2_O_2_ ↑ Methyl jasmonic acid ↓	Static liquid at 30 °C	18–110 U/mL (MnSO_4_)	[30]
	Gb_U87	JA ↑			
	Gb_35959	ammonium nitrate ↑ sodium nitrate↓ H_2_O_2_ ↓			
*T. pubescens*	MnP TP55	Mn^2+^ ↑	shake liquid at 30 °C	3.56 U/mg (2,6-DMP)	[4]
*P. ostreatus*	MnP4	Mn^2+^ ↑	shake liquid at 30 °C	1.2 U/mL (2,6-DMP)	[10]
*T. polyzona*	MnP1, MnP2	Light ↑	shake liquid at 30 °C	0.8–3.5 U/mL (2,6-DMP)	[31]
*Ceriporiopsis subvermispora*	MnP1, MnP2	Mn^2+^ and Cu^2+^ ↑	shake liquid at 30 °C	-	[32]
*Bjerkandera adusta*	-	Se^2+^ ↑	shake liquid at 28 °C	0.39–0.81 U/mL (MnSO_4_)	[33]
*Dichomitus squalens*	-	Mn^2+^ ↑	shake or static at 28 °C	-	[34]
*T. trogii*	T_trogii_09901, 09904, 09903 and 09906	Mn^2+^ ↑	Static liquid at 28 °C	2.9–15 U/mL (2,6-DMP)	This study
	T_trogii_11984, 11971, 11985 and 11983	Cu^2+^, H_2_O_2_ and temperature ↑	Static liquid at 28 °C		
	T_trogii_09901, 09904, 09903 and 11984	Cu^2+^ ↑	shake liquid at 28 °C	<0.55 U/mL (2,6-DMP)	

“-” indicated that no relevant data were provided in the cited references.

## Data Availability

The genome and putative mRNA sequencing data presented here are associated with NCBI BioProject PRJNA480364 and BioSample SAMN09635320. The datasets generated/analyzed during the present study are available and included either within this article or in the Supplemental Materials.

## Supplementary Materials

The following are available online at https://www.mdpi.com/article/10.3390/microorganisms9122595/s1, Figure S1: Exclusive domain prediction of TtMnPs. Table S1: PCR primer sets used for the gene cloning of the coding or promoter regions of TtMnP gene family. Table S2: Real time PCR primer sets used in this study. Table S3: The predicted and tallied physiochemical properties of 8 putative MnP genes in *T. trogii* S0301.
