# Supplementary material for: The Manganese Peroxidase Gene Family of Trametes trogii: Gene Identification and Expression Patterns Using Various Metal Ions under Different Culture Conditions
Source: Microorganisms. 2021 Dec 16;9(12):2595. doi: 10.3390/microorganisms9122595 (PMC8704794; doi:10.3390/microorganisms9122595)
Supplement: Supplementary file 1 [file microorganisms-09-02595-s001.zip › microorganisms-1489006-supplementary.pdf]

Table S1: PCR primer sets used for the cloning of the coding or promoter regions of TtMnP gene family.

Target Gene/Promoter	Primer	
	Name	Sequence (5′–3′)
T_trogii_09901	09901-for	ATGGCATTCAAAGCACTCGC
	09901-rev	TAAGAGGGAGGAACAGGGGC
T_trogii_09903	09903-for	TCGCGTCCTTTGTCTCTGTTC
	09903-rev	GTCATAGACGGGTCAGCGAA
T_trogii_09904	09904-for	CTGCCAACGGTGCTCTCATC
	09904-rev	GTCAGTGGGGAGAGTCGGGA
T_trogii_09906	09906-for	ATGGCCTTCAAGACTCTGGC
	09906-rev	CGACAGAAGTGACCGGACC
T_trogii_11971	11971-for	GTTCTCTATCGTCTCTTTGGCG
	11971-rev	AGGGAGTGTCAGCGCAAGC
T_trogii_11983	11983-for	ATGGCGTTCAAGGCTCTTCTCT
	11983-rev	TCAGAGATTGGGGACAGGCG
T_trogii_11984	11984-for	ATGGCTTTCAAAGCTCTGCTCTC
	11984-rev	AAGGTCGGGAAGGGGGTGT
T_trogii_11985	11985-for	CGCCCTCCAAGGTGCAA
	11985-rev	TAGCCGGACCAGGGTCAGTA
T_trogii_09901	09901-P-for	GACTCCCAATTCTTCATCGA
Promoter	09901-P-rev	GTCGCGTCAAGCCTTAACT
T_trogii_09903	09903-P-for	GTTCCGTAGCCGTGGTTCAG
Promoter	09903-P-rev	GAACAGAGACAAAGGACGCGA
T_trogii_09904	09904-P-for	CATCGAGACCCAGCTCCGT
Promoter	09904-P-rev	TGCTTTGAATGCCATGGTTG
T_trogii_09906	09906-P-for	CGGACCCTCCTTTTGTCTGATAG
Promoter	09906-P-rev	GAGAGGAGAGCTGTCGATGTCC
T_trogii_11971	11971-P-for	TCTGTGATCTGCTCCCTTGTC
Promoter	11971-P-ev	TGCTGATCGAGTGTTGAGGA
T_trogii_11983	11983-P-for	AGCCGCATATCCTCACGAGT
Promoter	11983-P-rev	AAGAGCCTTGAACGCCATTG
T_trogii_11984	11984-P-for	TAGACGTTCGTCTCAGCCCG
Promoter	11984-P-rev	GGCAGAGGCGTACCAATCG
T_trogii_11985	11985-P-for	ATTCCCGGCTGTCATAAACA
Promoter	11985-P-rev	TAGAGAGGAGAGCCTTGAACG

Table S2. Real time PCR primer sets used in this study.

Target gene	Primers	Primer sequences (5'-3')
TtGpd	TtGpd-S4	GGGCATTCTGGACTACACCGAGG
	TtGpd-A4	GCGATGAGCTTCACGAAGTTCTTG
*T_trogii*_11971	RT-11971-for	CGCCACAACATCTCAACTGCT
	RT-11971-rev	TGCGACCAAGGCGGAAC
*T_trogii*_09904	RT-09904-for	TGTCTCGAACTGCCCTGGTG
	RT-09904-rev	AGCGTTGGAGAATGCTGTCG
*T_trogii*_09906	RT-09906-for	CGGCACGGGTGGTAATCAG
	RT-09906-rev	GATGGTCATCTTGCGGAACG
*T_trogii*_11983	RT-11983-for	TCTGGCTCCTGTCGGCTCA
	RT-11983-rev	AAGTGGTCGGACTGGATACGC
*T_trogii*_11984	RT-11984-for	CGTTCCGCCTGACTTTCCA
	RT-11984-rev	GGCGATGATGGGCTTCTG
*T_trogii*_11985	RT-11985-for	TGCGAGTGGCAGTCGTTCA
	RT-11985-rev	GAACGACGGGAAGGGTGTGT
*T_trogii*_09901	RT-09901-for	CTGCCAACGGTGCTCTCATC
	RT-09901-rev	CGAAGGGATTCGTGGACCTC
*T_trogii*_09903	RT-09903-for	TGTTCTCGCTGCGTTCCAG
	RT-09903-rev	GCACCTCCACCACCAAATTG

Table S3. The predicted and tallied physiochemical properties of 8 putative MnP genes in *T. trogii* S0301.

Gene ID	Amino Acids	Molecular Weight (KDa)	pI	Signal Peptide Cleavage Site	Subcellular Localization Predicted
T_trogii_09901	364	38.40	4.44	21 - 22	extracellular
T_trogii_09903	364	38.64	4.34	21 - 22	extracellular
T_trogii_09904	364	38.43	4.44	21 - 22	extracellular
T_trogii_09906	358	38.21	4.89	21 - 22	extracellular
T_trogii_11971	365	39.04	4.32	21 - 22	extracellular
T_trogii_11983	367	39.31	4.85	21 - 22	extracellular
T_trogii_11984	364	38.94	4.94	21 - 22	extracellular
T_trogii_11985	358	38.28	4.79	21 - 22	extracellular

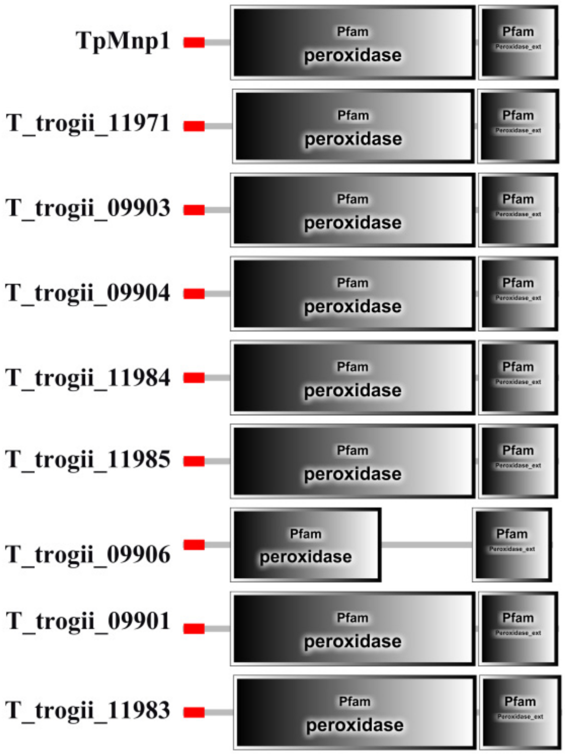

Figure S1. Exclusive domain prediction of TtMnPs. Exclusive domain prediction of TtMnPs. The domain architecture of TtMnPs were analysed by SMART software (http://smart.embl-heidelberg.de/), and the domain architecture of TpMnP1 (GenBank: BBB76257.1) was also displayed as an control. A total of three domains are predicted for each MnP, including signal peptide (marked red), Pfam:peroxidase and Pfam: Peroxidase_ext (marked Gray).
